# Direct Interstitial Treatment of Solid Tumors Using an Injectable Yttrium-90-Polymer Composite

**DOI:** 10.1089/cbr.2019.2947

**Published:** 2020-02-05

**Authors:** Darrell R. Fisher, Janean Fidel, Charles A. Maitz

**Affiliations:** ^1^ Department of Pharmaceutical Sciences, Washington State University, Spokane and Richland, Washington.; ^2^ Department of Veterinary Medicine, Washington State University, Pullman, Washington.; ^3^ Department of Veterinary Medicine and Surgery, University of Missouri, Columbia, Missouri.

**Keywords:** cancer treatment, radionuclide-polymer composite, sarcoma, therapeutic ratio, ^90^Y-radiogel, yttrium-90

## Abstract

***Purpose:*** Yttrium-90 (^90^Y)-polymer composite (radiogel) may be administered directly into cancerous tissues to deliver highly localized beta radiation for therapy. In a dose-escalation study, the authors investigated the feasibility of treating feline and canine soft-tissue sarcomas as a model for nonresectable solid tumors in humans to gain clinical experience and to identify optimal methods for placing the composite uniformly within target tumor tissue.

***Materials and Methods:*** Five cats (Washington State University) and three dogs (University of Missouri) were selected for treatment from among veterinary clinic patients presenting with subcutaneous soft-tissue sarcomas. The therapeutic radiogel composite comprised two parts that were combined before therapy: (1) a calibrated activity of highly insoluble ^90^Y(YPO_4_) particles in a sterile, phosphate-buffered saline solution and (2) a resorbable hydrogel delivery vehicle containing a dissolved copolymer of poly-(DL-lactic acid-co-glycolic acid) and poly-(ethylene glycol). Sarcomas of anesthetized animals (five cats and three dogs) were injected with the ^90^Y-radiogel (10%–15% by tumor volume) using a parallel-needle grid pattern with ∼4–5-mm spacings with or without ultrasound guidance. After injection, the composite solution gelled within tumor interstitial spaces to solid phase upon reaching body temperatures to constrain the ^90^Y activity intratumorally. The animals were then imaged with computed tomography (CT) or positron emission tomography (PET)/CT and placed in radiation isolation for overnight monitoring and follow-up.

***Results:*** Gelation of the composite within tumor extracellular spaces confined the ^90^Y(YPO_4_) particles in place to deliver a planned radiation absorbed dose (100–320 Gy) to target tissue through complete decay. Response of the tumor tissue to ^90^Y-radiation therapy postexcision was evaluated by imaging, tumor resection, and histology. Correlation was observed on histopathology between tumor destruction and radiation dose. With uniform placement at high dose, the authors achieved complete remission or stable disease (at 1–2 months posttreatment).

***Conclusions:*** This study demonstrated successful injection of ^90^Y-polymer composite (radiogel) without discernable radiation dose to normal organs or other detrimental side effects. Animal patients recovered quickly from the injection procedure. The better therapeutic responses were observed at mean doses at or above 300 Gy.

## Introduction

Cat and dog cancer patients with soft-tissue sarcomas were presented by their owners at the Washington State University (WSU) and University of Missouri (MU) veterinary hospitals for experimental cancer treatment. Both institutions are licensed to receive and handle yttrium-90 (^90^Y) and other radioactive materials. Investigators followed approved university procedures for animal welfare and for radiation safety as required by their respective Institutional Review Board, Institutional Animal Care and Use Committee (IACUC), and Radiation Safety Committee.

^90^Y-polymer-composite (radiogel) is a promising therapeutic agent that may be administered directly into cancerous tissues to deliver highly localized, high-dose beta radiation for therapy. Direct, uniform placement of ^90^Y-radiogel may provide a highly effective approach to complete tumor destruction without detrimental side-effects. Resulting high therapeutic ratios should be predictive of both safety and efficacy.

### Purpose

In a dose-escalation study, the authors treated feline vaccine-associated and canine soft-tissue sarcomas as a model^1^ for various nonresectable solid tumors in humans. The main objectives of this research were as follows: (1) to gain practical clinical experience administering and treating solid tumors with ^90^Y-radiogel, (2) to demonstrate ^90^Y-radiogel performance characteristics in tumor tissue after injection, and (3) to evaluate therapy effectiveness and monitor treated animals for potential adverse tissue reactions associated with localized high-dose therapy. The cat study was conducted first, starting with relatively low doses, to optimize methods for preparing and administering treatment. The dog study was performed later at higher doses to optimize therapy and to image the intratumoral biodistribution of the administered treatment.

Injectable ^90^Y-radiogel comprises an insoluble ^90^Y-yttrium-phosphate (YPO_4_) radiation source mixed within an injectable, thermally reversible, temperature-sensitive polymer solution. The mixture may be injected directly into tumor tissue. The mixture gels within tumor extracellular spaces after injection when it warms to body temperature.

Radiogel was designated as a medical device under the U.S. Food and Drug Administration (FDA) classification system. Radiogel incorporates commercially available, nontoxic pharmaceutical grade polymers, including polylactide, polyglycolide, and polylactic-co-glycolic acid copolymers. These well-known bioresorbable polyesters have wide applications in biomedicine and are FDA-approved for *in vivo* use in several drug and cosmetic products. Over time, natural breakdown products of radiogel include lactic acid and glycolic acid (also known as nontoxic natural byproducts of the Krebs cycle).

A typical formulation for interstitial implantation comprises a 25–30 weight percent solution of poly(lactic acid-co-glycolic acid)-g-poly(ethylene glycol), or PLGA-g-PEG, dissolved in sterile phosphate-buffered saline (PBS). The PLGA-g-PEG polymer solution has the consistency of slightly thickened water at or slightly below room temperature before injection. The polymer solution performs as a delivery vehicle for the active therapeutic component, microscopic ^90^Y(YPO_4_) particles. When implanted, the cold solution perfuses tumor extracellular space, warms to body temperature, and transitions (within a few seconds) to a solid-phase hydrogel. In the tumor, hydrogel helps to contain the ^90^Y radiation source through complete decay, and then later biodegrades and resorbs naturally over a period of about 2 months to nontoxic degradation byproducts.

Desirable radiogel properties for cancer treatment include material purity and sterility, simplicity of administration, intratumoral perfusion after injection to distribute the ^90^Y source material within the tumor, confinement of radioactivity to limit radiation dose outside the target tissue, lack of detrimental side effects of therapy, nontoxicity, and radiation safety aspects (defined by negligible or ultralow doses to the administering interventionalist and support staff).

A clinical advantage of beta radiation is the ability of oncologists to prescribe and deliver relatively high doses to the tumor while minimizing dose to adjacent (nontarget) healthy tissue. ^90^Y is a high-energy, β^−^-emitting radionuclide with no primary gamma emissions. The maximum energy in the ^90^Y β^−^-particle spectrum is about 2.3 MeV; ^90^Y has a mean energy of about 0.93 MeV.^2^ The maximum range of 2.3 MeV β^−^-particles in tissue is 11 mm, and the mean range of all emitted ^90^Y β^−^-particles in tissue is about 4.7 mm. ^90^Y decays with a half-life of 64 h to stable zirconium-90 in trace, nontoxic amounts. In cancer treatment, high-energy ^90^Y β^−^-particles cross-irradiate tumor tissue and help to overcome minor source distribution inhomogeneities.

In cats, feline vaccine-associated soft-tissue sarcoma was selected as a model tumor due to accessibility for injections. Feline sarcomas are difficult to treat using standard modalities; only a small percent of such cats (about 14%) receiving surgical treatment alone have long-term (>2-year) survival.^3^ Few treatment options are available for soft-tissue sarcomas once the tumors are established.^4^ Soft-tissue sarcomas in dogs may be excised surgically or treated using external-beam radiotherapy (42–57 Gy), with a median survival time of about 5 years when used in combination; however, side effects of external radiation therapy are common.^5^ Direct, localized radiotherapy using interstitially implanted ^90^Y(YPO_4_) microspheres enables significantly higher tumor doses without the detrimental side effects associated with external beam therapy.

Human clinical applications for ^90^Y-radiogel therapy could include nonresectable and radiation-resistant solid tumors that cannot be treated successfully using external beam radiation, systemic chemotherapy, surgery, or other modalities; such tumors may include certain brain, pancreas, liver, and potentially disfiguring head/neck tumors, among others.

### Therapeutic index

High therapeutic ratios are the objective of all cancer treatment; therefore, ^90^Y-radiogel was designed to provide the highest possible therapeutic index for treating nonresectable or radiation-resistant solid tumors *in vivo*. Therapeutic index (Ti) is the ratio of the radiation dose imparted to target cancer tissue (*D*tumor) and the dose-limiting normal tissue (*D*normal), such that

Ti=Dtumor∕Dnormal

The concept of therapeutic index incorporates concepts of both efficacy and safety by maximizing the effective radiation dose to target cancer while minimizing radiation dose to all other normal organs and tissues. Radiation dose to the target tissue may be maximized by (1) using a high-energy, pure beta-emitter, such as ^90^Y, (2) distributing the beta-emitter as homogenously as possible within the target tissue by injection and perfusion, and (3) confining the radioactive source to the target tissue.

## Materials and Methods

### Animal subjects

Private owners enrolled five cats (WSU) and three dogs (MU, Columbia) presenting with subcutaneous soft-tissue sarcomas for investigational therapy using ^90^Y-radiogel. Before treatment, the animal patients underwent complete veterinary physical examination, complete blood count, biochemistry, urinalysis, review of prior treatment, and disease staging. Tumor mass, shape, and position were determined from computed tomography (CT) imaging or caliper measurements for dosimetry and treatment planning.

### ^90^Y-radiogel composite

^90^Y-radiogel is a composite hydrogel comprising two sterile, apyrogenic solutions mixed together immediately before intratumoral injection: (1) a PBS solution containing a suspension of highly insoluble YPO_4_ particles with ^90^Y activity calibrated to date and time of injection and (2) an injectable, nontoxic, water-based polymer delivery solution (hydrogel) described below.

#### ^90^Y-phosphate particle solution

**FIG. 1.**
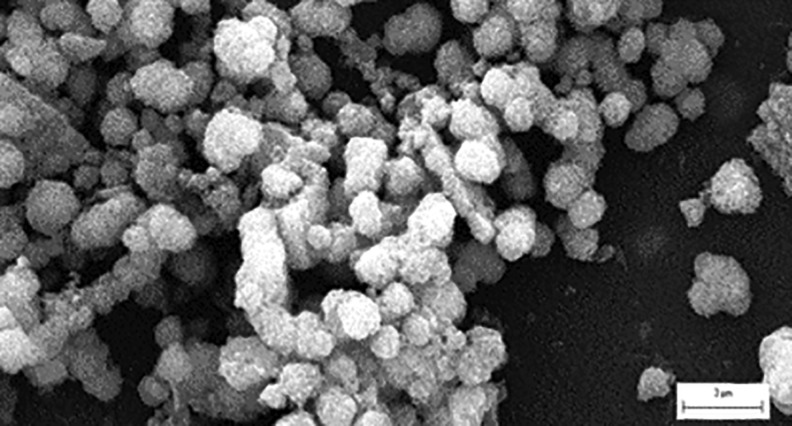
YPO_4_ crystalline particles (0.5–2 μm diameter, median diameter of 1.2 μm) imaged by electron microscopy with scale showing a marker scale length of 3.0 μm. YPO_4_, yttrium phosphate.

Calibrated amounts of ^90^Y were obtained from PerkinElmer (Waltham, Massachusetts) as a high-purity, ^90^Y-chloride radiochemical. ^90^Y(YPO_4_) particles (nearly monodisperse, nominally 0.5–2.0 μm diameter, Fig. 1) were prepared, assayed, analyzed, and checked for quality assurance and quality control by a certified laboratory (IsoTherapeutics Group LLC, Angleton, TX), washed free of unbound ^90^Y, and were suspended in PBS at ^90^Y activities and PBS concentrations predetermined by calculation to achieve a physician-prescribed activity and radiation absorbed dose to tumor tissue on day of implant (while maintaining a preferred polymer concentration at 25 weight%).

#### Synthesis of colloidal (^90^Y+^89^Y)PO_4_ microparticles

Highly insoluble YPO_4_ microparticles were synthesized using 0.1 M ethylenediaminetetraacetic acid (disodium EDTA), ultrapure water, high-purity 0.1 M yttrium chloride (^89^YCl_3_), 0.15 M sodium phosphate Na_2_HPO_4_), and a calibrated activity of ^90^YCl_3_ in 0.05M HCl, mixed in a container with Teflon liner and magnetic stirring bar. The acidity was adjusted to pH = 6.5 by adding 0.1 M HCl. The YPO_4_ microparticles were formed by controlled precipitation in a hydrothermal bomb or microwave oven at 150°C, followed by air-cooling with fast-stirring to avoid adhesion to container walls. The YPO_4_ microparticles were collected via vacuum filtration with ultrasonification, washed, and dispersed in PBS.

Product quality analyses were performed using X-ray diffraction to confirm presence of pure mineral xenotime (YPO_4_) as the major phase with halite (NaCl) as the minor phase. Microparticle sizing was performed using a MasterSizer 2000 (Malvern Instruments) coupled with a μP dispersion unit, and for the illustration (Fig. 1), physical diameters of the ^90^Y(YPO_4_) microspheres were confirmed by scanning electron microscopy (Fig. 1).

#### Dissolved polymer delivery solution

The delivery vehicle for the ^90^Y-phosphate particles is a sterile PBS solution containing a dissolved, nontoxic copolymer of PLGA and PEG at 25–30 weight%.

#### Composite hydrogel

Before injection, the hydrogel delivery solution was added to a small amount of PBS containing the calibrated ^90^Y activity within insoluble, high-purity YPO_4_ particles, and the composite was well-mixed using a magnetic stir bar. The mixture was cooled in ice before needle injection to enhance tumor perfusion and to slightly delay gelation after injection.

### Dosimetry for treatment planning

The Special Brachytherapy Modalities Task Group of the American Association of Physicists in Medicine (AAPM) recommended that medical internal radiation dose (MIRD)^*^ methods should be used for calculating radiation doses to organs and tissues from administered ^90^Y-microspheres.^6^ The MIRD formalism (Eq. 1) developed for radiopharmaceuticals used in both diagnostic imaging and cancer therapy^7^ may be correctly applied to placements of ^90^Y-microspheres in tumors, whether the ^90^Y microspheres are distributed homogeneously or nonhomogeneously.^8^ In the MIRD schema, the absorbed dose *D*(*r_T_*, *t*) to target tissue *r_T_* over a dose integration period *t* is as follows:

(1) $$D\left(r_{T},t\right)=\sum_{r_{S}}Ã\left(r_{S},t\right)S\left(r_{T}\leftarrow r_{S}\right),$$

where Ã(*r*_*S*_, *t*) is the time-integrated activity^†^ (i.e., total number of nuclear transformations) in source tissue *r_S_*, and where the value *S*(*r*_*T*_ ← *r*_*S*_) represents the mean absorbed dose rate to target region *r_T_* at time *t* after administration per unit activity present in source region *r_S_*. For injected tumors, the source and target regions are the same. The *S* value is determined specifically for ^90^Y emissions and the given geometry and density of the tumor.

$$\begin{array}{c} S\left(r_{T}\leftarrow r_{S},t\right)=\sum_{i}\Delta_{i}\Phi_{i}\left(r_{T}\leftarrow r_{S},E_{i},t\right)\\ =\sum_{i}E_{i}Y_{i}\frac{\phi_{i}\left(r_{T}\leftarrow r_{S},E_{i},t\right)}{m\left(r_{T},t\right)},\left(2\right) \end{array}$$

where *m*(*r*_*T*_, *t*) is the time-dependent mass of the target tissue, Δ_*i*_ is the mean energy emitted per radioactive decay or nuclear transformation, Φ_*i*_ is the energy absorbed fraction, and *E_i_* and *Y_i_* are the mean energy and yield of the *i*th radiation emitted per nuclear transformation in the radionuclide decay scheme. The specific energy absorbed fraction for ^90^Y beta decay may be determined using Monte Carlo electron transport or beta point-kernel calculations.

For a prescribed tumor dose (Gy) and known tumor mass, the appropriate radiogel injection volumes, ^90^Y activity, and material concentrations were calculated from Eqs. (1 and 2).^7,8^ Prescribed tumor doses in this study ranged from 100 to 320 Gy.

### Injection procedure

Five cats presenting with feline sarcoma and three dogs with soft-tissue sarcoma were shaved over the tumor, prepared, and anesthetized (desflurane or isoflurane) under standard surgical care.

Tumor surfaces of anesthetized subjects were disinfected and marked with regularly spaced injection points (about 4 to 5 mm apart) to facilitate placing the radiogel uniformly within the tumor boundaries and margin tissues. Using a parallel needle grid pattern, intratumoral injections were then made using standard 25 gauge needles and 1 cc or 3 cc syringes containing calibrated amounts (0.1–0.2 mL per point, or 10%–15% by tumor volume) of ^90^Y-radiogel under ultrasound guidance (five cats) or free-hand (three dogs).

Each injection was given using a continuous flow as the needle was withdrawn from the furthest point within the tumor to a point directly opposite. The skin was firmly opposed briefly after needle removal to promote gelation. Fine-gauge needles were preferred to minimize back leakage from the injection site, while still allowing free flow of the carrier hydrogel into the tumor. The injection site was then lightly wiped with an alcohol swab to remove any external ^90^Y contamination.

Upon injection, the composite solution carried the suspended ^90^Y particles into tumor interstitial fluid space where the solution perfused the target tissue radially. After warming to near body temperature (30–37°C), the dissolved polymer solution transitioned from liquid to gel phase,^9^ which solidified the mixture within the tumor tissue interstitium. In dogs, blood measurements for ^90^Y showed no significant ^90^Y activity postinjection; gelation entrapped ^90^Y particles in the tumor, preventing outmigration via blood circulation, and may have also blocked tumor interstitial fluid transport and respiration.

### Postinjection imaging, care, and observation

The tumors were imaged using standard CT or PET/CT^‡^ (Celesteion pureVision, Canon Medical, Tustin, CA) immediately after injection and again typically at 3 and 6 weeks postinjection to evaluate response to therapy. After the first imaging session, the animals were placed in isolation for overnight monitoring (or longer, if needed), medicated for pain (if indicated), and then released to owners.

Follow-up care included physical examinations, radiology, complete blood counts, biochemistry, tumor measurement, tumor biopsy, and postinjection tumor resection for histology and pathology to evaluate objective response to therapy. Bandages placed on some of the dogs limited the amount that they would lick at the injection site. The animals were checked for compliance to institutional release criteria before release to owners. Tumor response was evaluated using modified Eastern Cooperative Oncology Group (ECOG) performance criteria.

### Image analysis for placement dosimetry

The authors confirmed ^90^Y placement, postinjection biodistribution, and dosimetry using imaging system software in the three dogs. The dog PET/CT images were imported into a commercial radiation treatment planning software (RayStation; RaySearch Laboratories, Stockholm, Sweden), and anatomical structures of interest were delineated, generally including bone, lymph nodes, skin, and gross tumor volume. The contours were exported as a Digital Imaging and Communications in Medicine, a file format structure set and converted to NIfTI files via 3dSlicer.^10^ The PET/CT files and NIfTI files were evaluated on Philips Imalytics software (Philips GmbH Innovative Technologies, Aachen, Germany) using the *Y_90_4.42* algorithm with attenuation correction.

## Results

In this dose-escalation method development study, the authors delivered escalated absorbed doses of 100–320 Gy to target tissue (through complete decay), depending on the physician prescription. Overall, they observed dose-related objective tumor response to therapy. In the cats at lower doses, they observed no decrease in tumor volumes before surgical removal of the tumors. In dogs treated at higher doses, they observed complete response to treatment or stable disease.

### Intratumoral perfusion and biodistribution

^90^Y-radiogel was successfully administered intratumorally without premature gelation in the syringes or needles. Within about 15 s, the tumor tissues stiffened as the injection solution gelled interstitially. Gelation also minimized back-leakage of radiogel from the injection site.

**FIG. 2.**
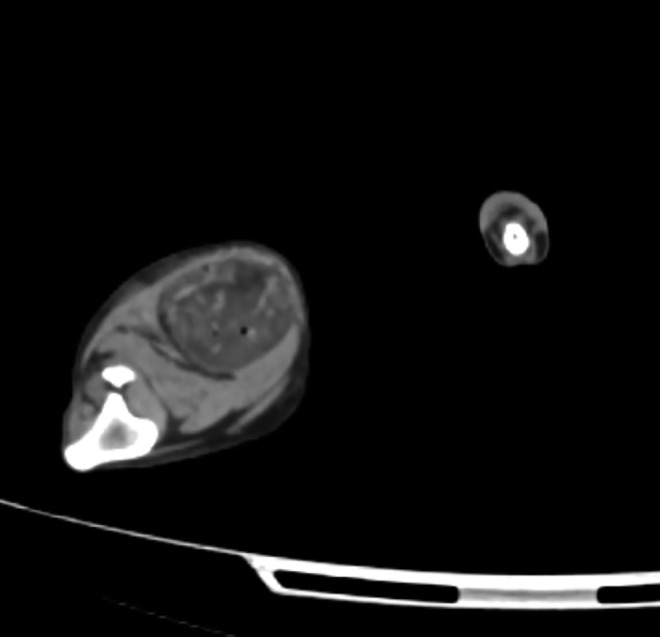
CT radiograph of a canine sarcoma in the hind limb of a dog treated with ^90^Y-radiogel (variegated darker mass in the *upper right*). The hyperattenuating material in the tumor relative to surrounding tissues indicates the potential imageability of elemental YPO_4_ placed into the tumor tissue by direct, interstitial injection. CT, computed tomography.

**FIG. 3.**
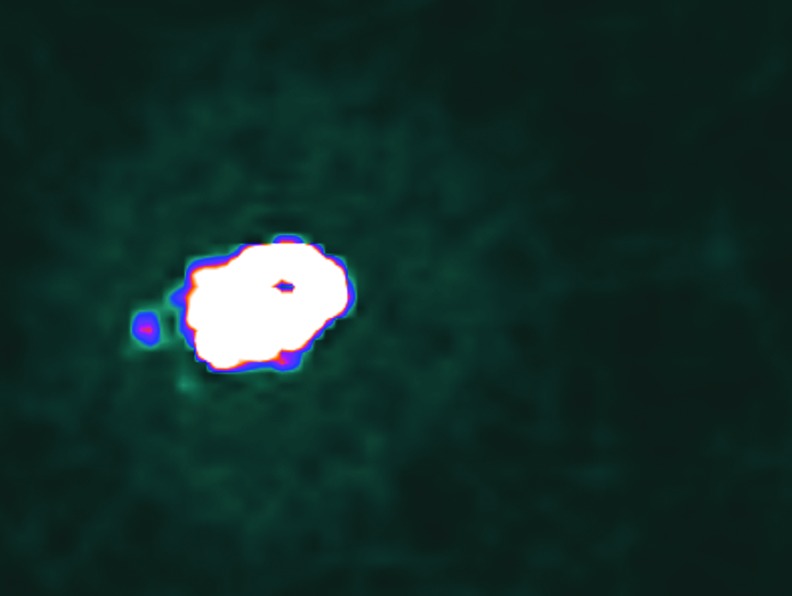
PET image of ^90^Y in the dog sarcoma showing a nearly uniform biodistribution of ^90^Y activity within the tumor and lack of ^90^Y counts recorded outside the tumor, indicating successful placement of ^90^Y-radiogel. PET, positron emission tomography; ^90^Y, yttrium-90.

**FIG. 4.**
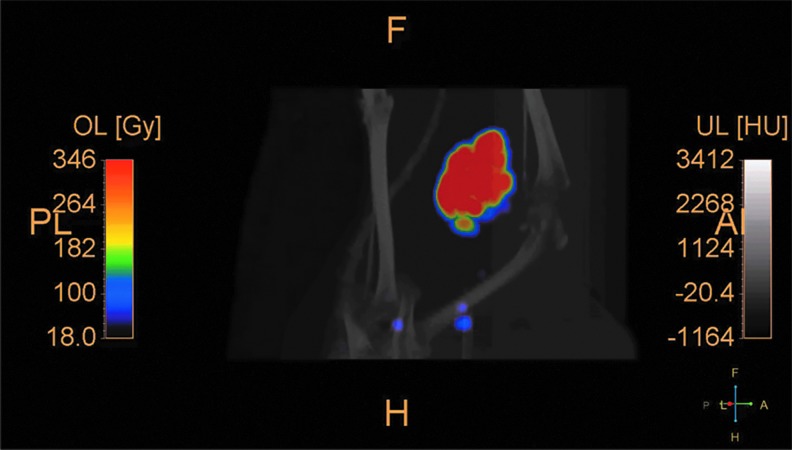
PET/CT coregistered image at about 30 min postinjection, confirming placement of ^90^Y-radiogel in the canine soft-tissue sarcoma; no evidence of activity migration away from the tumor is visible in this image. The color scale shows isodose curves as percent (ordinate) less than absorbed dose in Gy (abscissa).

The biodistribution of ^90^Y sources in dog sarcomas was imaged by positron-emission tomography. Figure 2 shows a CT radiographic image of a canine soft-tissue sarcoma in the right hind limb about 30 min after treatment with ^90^Y-radiogel. Figure 3 shows the PET image of the tumor. Figure 4 shows the coregistered PET/CT images of the same canine tumor. Figures 2–4 show that the ^90^Y(YPO_4_) particles perfused tumor tissue after injection and distributed via extracellular fluid space in a manner that provided a homogenous radiation dose to most of the tumor mass. Imaging did not show bolus hydrogel deposits or columnar hydrogel formations in the tumor associated with needle injections.

**FIG. 5.**
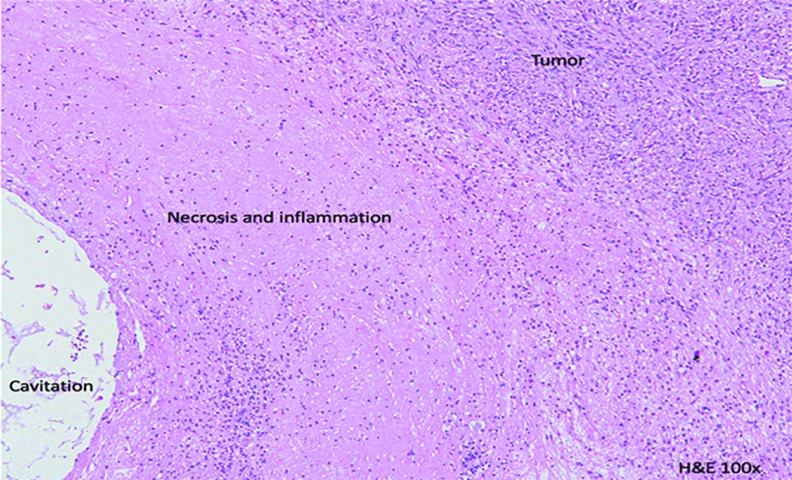
Radiation-related tumor necrosis and inflammation in a H&E-stained histology section obtained from a cat treated for feline sarcoma with ^90^Y-radiogel. H&E, hematoxylin and eosin.

**FIG. 6.**
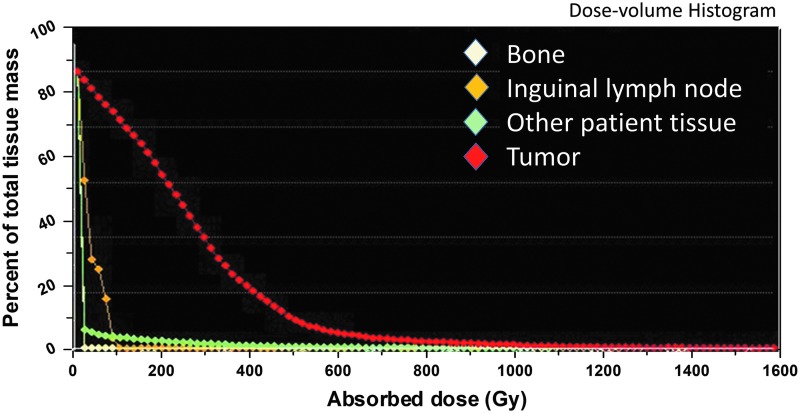
Dose-volume histogram calculated by the PET Imalytics software for neighboring bone, inguinal lymph node, patient whole body, and tumor tissue. Therapeutic index may be calculated as the ratio of the areas-under-curve for tumor (*dotted red curve*) relative to other normal tissues within the same PET image.

A sectioned feline tumor slice stained with hematoxylin and eosin (Fig. 5) shows tumor necrosis and inflammation associated with therapy provided by ^90^Y-radiogel. A dose-volume histogram from a dog tumor (Fig. 6), based on gross tumor volume, geometry, and measured ^90^Y activity in the tumor (and surrounding tissues), provided strong evidence for uniform radiogel placement. These images showed that gelation of the radiogel polymer composite within tumor extracellular spaces held the ^90^Y(YPO_4_) particles in place; a trace amount of drainage to a nearest lymph node was observed in one treated dog, demonstrating that the ^90^Y(YPO_4_) activity distributed interstitially rather than vascularly.

PLGA copolymers represent a family of FDA-approved biodegradable polymers that are physically strong and highly biocompatible. PLGA is most popular among the various available biodegradable polymers because of its long clinical experience and favorable degradation characteristics. During injection, the biodegradable PLGA/PEG polymer solution serves first as the ^90^Y(YPO_4_) delivery carrier and second as a molecular scaffold for confining the ^90^Y(YPO_4_) microparticles within the tumor interstitium; gelation effectively blocks extracellular fluid transport and precludes ^90^Y-microparticle outmigration from the tumor. Over time (4–8 weeks), the gel resorbs naturally by syneresis to nontoxic breakdown products.

### Tumor response to treatment

Table 1 shows the therapy administered and the response in five cats and three dogs. Response to ^90^Y radiation therapy was evaluated by pathology and histology for all feline tumors postexcision. Definitive correlation was observed on histopathology between tumor cell killing and sites of ^90^Y-radiogel placement.

**Table 1.** Cat and Dog Patients Treated with Yttrium-90-Polymer Composite (Radiogel)

	Tumor type	Starting tumor mass (g)	Prescribed tumor dose (Gy)	^90^Y administered activity (mBq)	Clinical responses
Cat 1	Fibrosarcoma, lateral thorax	28.8	100	68	Irregular placement; objective response observed on histopathology by profound cell killing in tumor regions where radiogel was delivered. Outwardly the tumor did not respond to injections. Tumor was removed at 6 weeks postinjection with clean, narrow margins; follow-up radiation with 30 Gy was given. Cat is still alive at 30 months.
Cat 2	Feline vaccine-associated soft-tissue sarcoma	100	160	377	Uniform placement; objective tumor response observed on histopathology. No side effects, but the cat was euthanized at 3-weeks post-therapy due to kidney failure unrelated to treatment (a pre-existing kidney disease)
Cat 3	Fibrosarcoma, lateral thorax and ribs	96.3	300	680	Uniformly placed with partial tumor response and necrosis on histopatholgy but no decrease in volume. The cat was euthanized at 6 weeks due to a resistant infection postsurgery unrelated to radiogel treatment; no side effects of therapy were observed.
Cat 4	High-grade feline soft-tissue sarcoma, lateral thigh	118	300	777	Irregular radiogel placement due to tumor skeletal involvement and advanced-stage tumor progression; partial response in treated areas and no adverse effects of therapy. Required surgical excision and 30 Gy external beam; cat well at home 18 months postinjection.
Cat 5	Aggressive fibrosarcoma at site of prior limb amputation	78.6	300	566	Uniform placement with some radiogel leakage due to incorrect mixing; no objective response to therapy, but survived 5 months postinjection.
Dog 1	Soft-tissue sarcoma	67	300	474	Complete response (fully healed at 3 months posttherapy). Edge-of-field recurrence at 8 months posttreatment. Treated again with ^90^Y-radiogel (255 Gy, results pending).
Dog 2	Soft-tissue sarcoma	1.14	320	10.4	Complete response, no discrete mass.
Dog 3	Soft-tissue sarcoma	233	200	1100	Stable disease and follow-up continues.

In this dose-escalation study, the specific aim of the cat injections was to investigate and develop best approach and methods. The specific aim of the dog injections was to apply those methods and successfully destroy the tumor mass.

90 Y, yttrium-90.

Two cats with tumors that could be removed were alive at 32 and 17 months posttreatment. Three cats with tumors removed were alive up to 26 months posttreatment. In one dog with a 67 g sarcoma treated at 320 Gy, the authors observed a complete response and healing at 3 months postinjection. Eight months after treatment, this patient had tumor recurrence at the periphery of the previously treated site and was treated with a second course of ^90^Y-radiogel (255 Gy). A second dog with a small 1.1-g sarcoma treated at 300 Gy also showed complete response after therapy; no discernable mass could be identified at 1 month posttreatment, and only an area of indiscrete swelling was noted. The third dog with a large (233-g) sarcoma had stable disease at 1 month. Patient follow-up continues.

### Side effects of therapy

^90^Y-polymer composite (radiogel) remained in tumors and ^90^Y did not migrate from the tumor to the major normal organs or bone marrow. No evidence was observed of any significant (detectable) ^90^Y activity entering blood circulation and depositing in any other organ or tissue.

Postinjection, the cats exhibited some pain in the tumors, and all had decreased appetites for 1–2 days postinjection. Tumors were removed for examination; two involved successful surgeries where clean margins were attained. Follow-up external beam radiation was given (30 Gy) due to the small volumes of margins attained. One cat was euthanized at 3 weeks due to health concerns unrelated to treatment, and the tumor was harvested for examination. One cat had successful surgery to remove the tumor, but developed a resistant infection postsurgery and was euthanized. One cat that had been heavily pretreated with surgery and chemotherapy was euthanized due to tumor growth at 5 months postinjection.

Dogs had variable skin side effects of treatment (Veterinary Radiation Therapy Oncology Group grade 1–2). The two dogs with large tumors developed draining tracts 3–4 weeks after treatment, presumably due to tumor necrosis. The tumor in the first dog completely involuted and the tumor site healed 6 weeks later.

## Discussion

In general, prognosis is excellent for dogs with soft tissue sarcoma that can be excised with wide margins or consolidated with external beam radiotherapy after removal of gross tumor. However, some animal patients are not good surgical candidates, and external beam radiation in the presence of gross disease is generally unrewarding. Depending on specific circumstances, resection of soft-tissue sarcomas can have other debilitating effects on patient quality of life. In this case series, sarcomas were selected as a model tumor for lesions that could be treated safely *in vivo* using an interstitially administered, high-dose ^90^Y-radiogel.

As a first-in-animal clinical study, the research plan was designed to gain practical clinical experience, identify and test optimal methods for placing the therapeutic agent within target tissue, characterize the behavior of ^90^Y-radiogel in the tumor after injection, demonstrate imageability postinjection, and evaluate biological response to therapy. Testing in veterinary patients (five cats, three dogs) confirmed that ^90^Y-radiogel can be placed interstitially in target tissue (tumors and margins) to achieve an effective, high-dose (100–320 Gy) therapy. Therapy involved multiple parallel injections (one at a time) under anesthesia, with or without ultrasound.

An absorbed dose of 300 Gy can be achieved by administering about 550 MBq (15 mCi) ^90^Y-radiogel into a typical 65 g tumor mass, for which the ^90^Y energy-specific absorbed fraction is about 0.85. Energetic ^90^Y β^−^-emissions made it possible to overcome some of the placement inhomogeneities inherently associated with an injectable delivery solution. Higher tumor doses may be achieved by increasing the activity of the administered therapy. With intratumoral ^90^Y-radiogel therapy, the radiation dose to any other normal organ or tissue is negligible.

Elemental YPO_4_ is highly insoluble; the crystalline structure of YPO_4_ lends to high chemical stability such that it is unlikely for ^90^Y atoms to dissolve from the particles and migrate into circulating blood. In this experience, the authors found that it was not likely to inadvertently introduce ^90^Y-radiogel into blood vessels by injecting solid tumor tissue. The hydrogel solidified after injection, which helped to confine the ^90^Y(YPO_4_) particles interstitially by preventing redistribution to the circulatory system to other organs and tissues of the body. They did observe a small amount of (expected) drainage of the administered YPO_4_ from interstitial fluids into the lymphatic system of one dog without adverse consequence.

Prior studies (unpublished) at the laboratory showed that elemental YPO_4_ injected into normal tissue is sufficiently dense to be imageable using conventional X-ray or CT radiography. The ability to image an injection of ^90^Y(YPO_4_) using both CT (contrast density) and PET (positron annihilation) should prove useful in future studies for confirming ^90^Y-radiogel placement postinjection.

### Dose-volume histogram and therapeutic index

Positron-emission tomography recorded the locations of radioactive source material within tissue. A dose-volume histogram was calculated using the Philips Imalytics (Koninklijke Philips N.V, Amsterdam, The Netherlands) software for neighboring bone, inguinal lymph node, patient body, and tumor tissue (Fig. 6). Due to the finite ranges of high-energy beta particles in tissues, actual radiation doses imparted to the tumor would be represented by a smoothed dose-volume histogram (not shown).

One representation of therapeutic index is the ratio of the areas-under-curve for tumor relative to other normal organs in a dose-volume histogram that may be considered critical, dose-limiting tissues. Exact values of therapeutic ratio could not be determined for red marrow, liver, kidneys, or even adjacent skin surfaces, because the authors did not see ^90^Y activity in these tissues; however, they estimate from these results the therapeutic ratios of 1000 or more for organs and tissues other than adjacent skin.

This first-in-animal clinical study showed feasibility of treating sarcomas and potentially many other solid tumors using an injectable ^90^Y-radiogel. The advantages are high therapeutic ratios with ^90^Y activity confined to target tumor tissues and negligible activity outside the target mass, resulting in objective response or complete response without adverse side effects. Tumor response to therapy may be size-dependent. Each of the cat tumors was very large at time of treatment, and treatment produced stable disease. One dog tumor that was large had stable disease after therapy, but not complete disappearance. Larger tumors correlated with longer disappearance times and formation of draining tracts. Follow-on treatment with ^90^Y-radiogel is not contraindicated and could provide additional benefit for treating persistent disease.

From this study, the authors (1) gained relevant experience, (2) identified successful injection techniques, (3) learned to achieve a uniform placement, (4) observed material behavior that matched expectations, (5) demonstrated radiation safety, (6) demonstrated usefulness of ultrasound-guided needle placement in the tumor, (7) observed fast and full recovery of the patients after injection therapy under anesthesia, and (8) identified best methods for preparing and mixing solutions, minimizing contaminations, selecting best needle sizes, and injecting the ^90^Y-radiogel. The rate of *in vivo* gelation was partly controlled by cooling the injection materials in ice before injection. In this study, they experienced no premature gelation within syringes or needles.

Some of the animal patients were treated as an experimental exercise, even though tumors had reached a late-stage massive size. Even at less-than-optimum doses, they observed tumor-tissue destruction associated with ^90^Y placement, therapy-related tumor necrosis, and in most cases, an acceptable treatment side effect profile.

## Conclusions

^90^Y-radiogel was designed to provide the highest possible therapeutic index for treating nonresectable or radiation-resistant solid tumors *in vivo*. PET/CT imaging showed that ^90^Y-radiogel could be administered without significant outmigration of ^90^Y-particles to normal organs and tissues. PET/CT confirmed that injected ^90^Y(YPO_4_) particles perfused tumor tissue in a manner that provided a homogenous radiation dose to most of the tumor mass. The authors' experience confirmed that ^90^Y-radiogel can be prepared and administered safely for therapy of nonresectable solid tumors in man and animals, including deeply seated tumors accessible via needle as well as tumors at or near skin surfaces. Feline vaccine-associated sarcoma and canine soft-tissue sarcoma serve as excellent models for testing ^90^Y-radiogel properties, safety, and clinical efficacy. Microscopic extension of sarcomas or metastatic migration into surrounding tissues may limit this model, as the therapy is mainly designed to treat gross disease; microscopic disease extension was likely the reason for edge-of-field recurrence in the first dog, which was later retreated.

Performance of the ^90^Y-radiogel met or exceeded design expectations. After injection, the ^90^Y- hydrogel composite solution gelled within interstitial spaces upon reaching body temperatures to contain the ^90^Y activity intratumorally. ^90^Y-phosphate particles remained in treated tumor tissue through complete decay without migrating vascularly to any normal organ or tissue.

Treated cats and dogs experienced no radiation-related illness associated with interstitially placed ^90^Y-radiogel therapy. The authors observed no adverse tissue reactions in any adjacent or distal normal organ or tissue beyond adjacent skin. With subcutaneous tumor tissue receiving an absorbed dose of about 320 Gy or less, they observed only mild skin erythema (reddening) with tumors in close contact with skin, and minor skin irritation in a few cases, partly due to brief surface contamination (easily removed with an alcohol wipe). In one dog, the draining tract healed completely within 6 weeks. In another dog, the nonhealing wound persisted for 4 months, after which surgical debulking was advised.

As seen on histopathology, tumor tissues responded well to treatment, with strong evidence of tumor cell killing associated with localized radiation dose. Animal subjects recovered quickly from the injection procedure. With uniform placement at high dose, the authors achieved complete remission or stable disease (at 1–2 months posttreatment). These results confirm the substantial opportunity for using ^90^Y-polymer composite (radiogel) to treat solid tumors in both human and veterinary patients.

## Notes

^*^ Special committee on Medical Internal Radiation Dose (MIRD) of the Society of Nuclear Medicine and Medical Imaging, Reston, VA.

^†^ This quantity was previously called *cumulated activity*.

^‡^ Yttrium-90 emits low-abundance positrons (about 32 per million decays) that may be imaged using positron-emission tomography. The Toshiba PET/CT is housed within the University of Missouri's Small Animal Hospital.
